# Carbonized Leather Waste: A Review and Conductivity Outlook

**DOI:** 10.3390/polym15041028

**Published:** 2023-02-18

**Authors:** Jaroslav Stejskal, Fahanwi Asabuwa Ngwabebhoh, Petr Sáha, Jan Prokeš

**Affiliations:** 1 University Institute, Tomas Bata University in Zlin, 760 01 Zlin, Czech Republic; 2 Faculty of Mathematics and Physics, Charles University, 180 00 Prague 8, Czech Republic

**Keywords:** leather waste, carbonization, pyrolysis, conductivity, nitrogen-containing carbon, char, activation

## Abstract

The carbonization of collagen-based leather waste to nitrogen-containing carbon is reviewed with respect to the preparation, characterization of carbonized products, and applications proposed in the literature. The resulting nitrogen-containing carbons with fibrous morphology have been used as adsorbents in water pollution treatment, in electrocatalysis, and especially in electrodes of energy-storage devices, such as supercapacitors and batteries. Although electrical conductivity has been implicitly exploited in many cases, the quantitative determination of this parameter has been addressed in the literature only marginally. In this report, attention has been newly paid to the determination of conductivity and its dependence on carbonization temperature. The resulting powders cannot be compressed into pellets for routine conductivity determination. A new method has been used to follow the resistivity of powders as a function of pressure up to 10 MPa. The conductivity at this pressure increased from 9.4 × 10^−8^ S cm^−1^ for carbonization at 500 °C to 5.3 S cm^−1^ at 1000 °C. The conductivity of the last sample was comparable with conducting polymers such as polypyrrole. The carbonized leather thus has the potential to be used in applications requiring electrical conduction.

## 1. Preamble

The carbonization of leather waste has two facets. The first, environmental, is represented by the conversion of the waste to products that can be further applied in various directions. The second concerns the preparation of new nitrogen-containing carbons. The carbonaceous materials are used as economic adsorbents valued in water-pollution treatment and their conductivity is of importance for the design of electrodes in energy conversion and storage. The reprocessing of leather waste based on the conversion of collagen biopolymer to carbons is reviewed. So far only marginal attention has been paid to its electrical properties. The conductivity of leather waste carbonized at various temperatures has been shown to reach the level of organic conducting polymers.

## 2. Leather Waste

The sustainable circular economy is oriented toward the reduction of waste and its recycling or conversion to new materials [1]. For example, it is estimated that the leather processing industry produces more than a hundred times more waste than total product output [2]. Leather waste treatment has recently been reviewed [2,3,4], with carbonization to nitrogen-containing carbons being so far investigated only to a limited extent as one of the feasible ways to convert the waste to potentially useful products [5,6,7]. The waste results most often from the processing of bovine skins/hides [8,9,10,11,12] and also hides from other animals, e.g., goats [13,14], pigs [6,13], rabbits [15], or sheep [13]. In many studies, however, the source has not been identified. The leathers were vegetable-tanned [14] but mainly chromium-tanned in various ways [12]. The leather waste studied in the literature thus has various origins and ways of treatment and, for this reason, a quantitative comparison of the reported data is difficult; the general trends, however, are clearly visible.

## 3. Carbonization

### 3.1. Temperature

**Figure 1:**
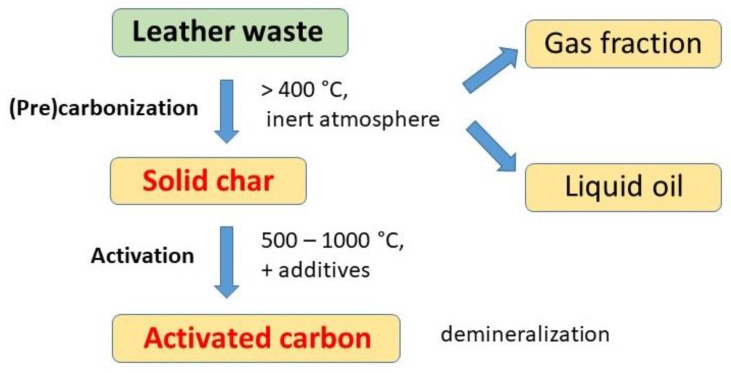
The carbonization of leather wastes yields solid char, liquid oil and gas fraction. Solid char is activated to nitrogen-containing carbon.

In simple carbonization, the crushed or powdered leather waste is heated at elevated temperatures in an inert nitrogen atmosphere to obtain a solid char (Figure 1). In some studies, the temperature is relatively low. Hydrothermal carbonization has been carried out in aqueous mediums at temperatures between 100–200 °C [16,17] and even temperatures below 200 °C produced materials with activity in the photocatalytic decomposition of water pollutants [15]. Higher temperatures, 400–500 °C, have been used more often [18] and the carbonizations have been usually carried out above 500 °C [19,20,21], up to 1000 °C [22]. Still, higher temperatures have not been used because of the low yields expected under such conditions.

The carbonization as a rule proceeds in an electrically heated oven but some atypical cases are mentioned in the literature. For instance, the carbonization of leather by microwave heating in inert atmospheres has been applied [23], and that occurring during laser cutting has also recently been investigated [14,24].

The majority of experiments involve leather waste as a single carbonization precursor. Only exceptionally has the pyrolysis of leather-containing composites with polyurethanes [25], or the conversion of a freeze-dried milled leather/poly(vinyl alcohol) composite to a carbonaceous aerogel [26] been reported. The joint treatment of leather tannery waste with hardwood pellets falls also into this category of experiments [27].

### 3.2. Carbonization Products

**Figure 2:**
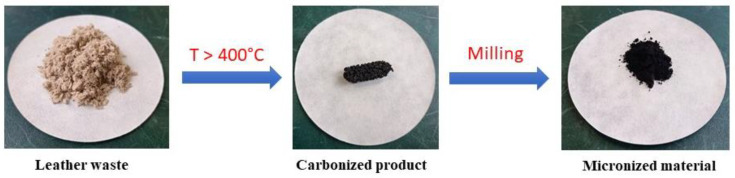
The shredded leather waste is carbonized at elevated temperatures and the product is homogenized by milling.

The carbonization of leather wastes generally produced three fractions—a solid char, a condensable liquid oil/tar, and a gas fraction [10,28,29,30,31,32,33,34] (Figure 1). The present review concentrates only on the solid product, also referred to as biochar (Figure 2). The pyrolysis conditions—rate of heating, target temperature, time of exposure and type and flow of inert gas—have been optimized with respect to yields. The three products, char, oil, and gas, are about balanced at 550 °C [35] and the completion of pyrolysis has been found at 650 °C [36]. Both the pyrolytic gas and liquid oil are a multi-component mixture of hydrocarbons and derived nitrogen-containing organic compounds [9,33]. The char consists mainly of nitrogen-containing carbon [37] but also a significant fraction of inorganic compounds, e.g., chromium-based from the tanning, or potassium salts resulting from the activation treatment.

### 3.3. Thermogravimetric Analysis

At an analytical level, the yield of char can be estimated from the thermogravimetric analysis in an inert atmosphere [9,13,38,39,40]. This is of importance for the planning of the preparative scale. Thermogravimetry has sometimes been coupled with FTIR spectroscopy [11] or mass spectrometry to identify the volatile decomposition products [13,41]. The results have been used for the formulation of pyrolysis models [40,42,43]. The starting transformation of collagen structure takes place already in the interval 100–240 °C [11]. Thermogravimetric analysis has indicated that different patterns can be obtained for leathers of different origins [13] but the main features of carbonization were preserved. Mathematical models of the pyrolysis kinetics have been proposed and illustrated on the waste from tanned cow skin [8], alkali-treated chrome-tanned [38] and vegetable-tanned leather [44]. The kinetics of pyrolysis has also been analysed for different heating rates [45] and with the help of thermogravimetric analysis [43]. The pyrolysis has been catalysed by cobalt or manganese chloride, which leads to a decrease in decomposition temperature [46]. We can speculate that the chromium compounds present in tanned leathers may have a similar effect.

### 3.4. Yield

The yield of char is one of the important parameters that determine the profitability of the preparation. The carbonization temperature should be high enough to assure the conversion of the starting biomaterial to a carbonaceous product. On the other hand, any increase in the temperature is associated with a decrease in the biochar yield and an increase in the cost of energy consumed in the process. For example, chromium- and vegetable-tanned shavings wastes have been exposed to elevated temperatures in inert atmospheres, leaving the 38–49 wt% char at 450 °C and 32–44 wt% at 600 °C [33,47,48]. The char yield was between 30–35 wt% at 750 °C [9] and various leathers left the char residue 20–30 wt% at 900 °C [49]. The main mass reduction takes place below 500 °C and the yield decreased only moderately above this temperature. All authors generally agree that the char yield becomes reduced with increasing carbonization temperature and the yield >20 wt% is still economically acceptable at temperatures even close to 1000 °C.

The preparation sometimes includes the demineralization step, i.e. the removal of inorganic salts, such as carbonates, chromium or potassium compounds, etc. [50]. In this procedure, the char is treated with, for example, 10% hydrochloric acid at 100 °C followed by repeated washing with distilled water [47,48,51]. This step reduces the final yield.

### 3.5. Activation

The char obtained by carbonization has often served as a precursor for the preparation of activated carbon. The activation is based on the heating of the substrate with activating agents still in an inert atmosphere. Such a process is meant to degrade the residual organic structures and generally leads to an increase in the specific surface area and porosity that is favourable for applications based on adsorption phenomena. The chars obtained at the precarbonization step at a lower temperature, e.g., below 600 °C are activated at a higher temperature. For example, the demineralized char has been activated for 4–10 h at 900 °C by flowing carbon dioxide [47,48,51,52] or steam [53]. Activation with potassium hydroxide at various temperatures is the most common [20,37,54,55,56,57,58,59,60,61,62,63]. The activation agents have an alkaline nature, e.g., sodium hydroxide [64], calcium carbonate [60,65], potassium carbonate [57,61] or an acidic one, e.g., zinc chloride [51,66] or pyrophosphoric acid [7,67]. The activation is associated with an additional reduction of mass. Precarbonization is sometimes carried out in air [68]. A single-step activation without precarbonization has also been reported [54,61,65].

## 4. Characterization

### 4.1. Morphology

**Figure 3:**
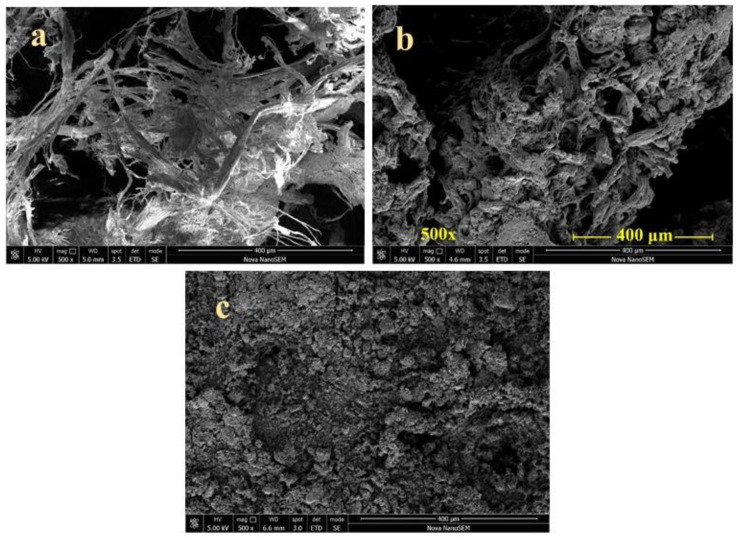
SEM micrographs of (**a**) fibrous leather waste, (**b**) carbonized product and (**c**) powdered material.

From a macroscopic point of view, the carbonized products are obtained as aerogels [69] that can be easily disintegrated into powders. The microstructure based on collagen fibres observed by scanning electron microscopy is preserved after carbonization except for some fusing shrinkage (Figure 3) [33,51,56,61,68,70,71]. High-resolution transmission electron microscopy has revealed graphitic nanolayers in onion-like nanomorphology of leather carbonized at 1000 °C [22] or a nanoribbon-like morphology [72]. The presence of chromium nitride nanoparticles has also been detected by transmission electron microscopy [68].

### 4.2. Nitrogen Content

Leathers are chemically composed of collagen polypeptide, which contains, in contrast to other biopolymers like polysaccharides, nitrogen atoms [8,10,56,60] at the C/N ratio ≈4–7. The carbonization thus leads to the nitrogen-containing carbons [10,37,57,62,63,73]. In this respect, this is similar to the carbonization of conducting polymers, such as polyaniline and polypyrrole [74,75]. The presence of several per cent of nitrogen atoms, and specifically a lone electron pair on the nitrogen atom, has importance for interactions with various species that manifest themselves in adsorption phenomena or (electro)catalytic performance [56]. Among them, hydrogen bonding between nitrogen and hydrogen atoms is probably the most important type of interaction.

### 4.3. Chromium Content

Chromium is an element closely associated with leather processing by tanning and toxicity [76,77]. When chromium-tanned leathers are carbonized, resulting carbons contain chromium in various oxidation states [33,50,66] with dominant chromium(III) oxide nanoparticles of 50–200 nm size [32,51,62,66,78]. The typical content of chromium oxides in char is several wt% [50], which increases after activation and may then exceed 10 wt%. Chromium(III) oxide is not toxic due to its insolubility in water but Cr(III) species are accompanied by harmful Cr(VI) ones [50,79], especially if oxygen traces or oxidants are present at high carbonization temperatures. Depending on the carbonization process, however, the presence of Cr(VI) in the products can be completely eliminated [33,50,71]. Chromium nitride nanoparticles have been observed by transmission electron microscopy [68]. The carbonized leathers also contain a non-negligible fraction of other inorganic elements, represented by iron, cobalt, nickel [22], calcium, magnesium, various trace elements, and especially potassium, when the corresponding hydroxide is used for the activation.

### 4.4. Specific Surface Area

The specific surface area is of importance for applications such as adsorbents. This parameter is low for untreated chars, <10 m^2^g^−1^ [15], of the order tens m^2^g^−1^ at the best [51,78], and only after the activation is increased to hundreds m^2^g^−1^ [7,20,55,66]. This parameter also increases after demineralization, e.g., from 428 to 927 m^2^g^−1^ [52], when the removal of soluble inorganics makes more pores accessible.

In addition to temperature, the specific surface area also depends on the type of activation agent and time spent at an elevated temperature. For example, leather pyrolysed at 800 °C had a specific surface area 47 m^2^g^−1^, which increases after activation in carbon dioxide at 850 °C to 889 m^2^g^−1^ [51,78]. The highest specific surface area of activated chars prepared at 450 °C is 799 m^2^g^−1^ and 295 m^2^g^−1^ for preparation at 650 °C [47]. Other samples carbonized at 450 °C have had a specific surface area close to 420 m^2^g^−1^ after various activations. This parameter is often reported to exceed 1000 m^2^g^−1^, e.g., 1602 m^2^g^−1^ [54], 1660 m^2^g^−1^ [61], 1664 m^2^g^−1^ [57], 2100 m^2^g^−1^ [56], 2247 m^2^g^−1^ [60], 2523 m^2^g^−1^ [69], and 3211 m^2^g^−1^ [59]. The associated porosity and pore-size fraction have also been reported and follow the same trend [21,57].

### 4.5. Spectroscopy

Raman spectroscopy is a powerful tool to follow molecular changes during the carbonization process. The spectra of carbonized leathers display two characteristic peaks located at ca. 1560 and 1350 cm^−1^ assigned to the graphitic and disordered structures, respectively [7,22,56,63,80]. Such spectra have been observed in virtually all carbonized organic materials [81], but the ratio of their intensities, and thus proportions of the microstructures, differ. As the carbonization temperature increases, the conversion of Raman spectra of the original leather to above twin-band patterns has been observed.

The evolution of FTIR spectra as a function of carbonization temperature has been reported less often [7,82] and the gradual disappearance of the bands corresponding to the individual bond vibrations has been observed [81,83].

### 4.6. Electrochemistry

Electrochemical characterization can be found, especially in papers associated with the design of supercapacitors. These have included cyclic voltammetry, galvanostatic charge–discharge measurements and electrochemical impedance spectroscopy [7,20,55,59,63]. These types of experiments implicitly assume a certain level of electrical conductivity, which is dealt with separately below.

### 4.7. Magnetic Properties

No magnetism is expected in carbonized organic material. Nevertheless, magnetic features have been reported and the saturation magnetization of leather carbonized at 1000 °C for 8 h has reached 5 emu g^−1^ [22]. The origin of magnetism is likely to arise from inorganic ferromagnetic impurities. Various chromic oxides and compounds like magnesium dichromate found in the char [50] are the candidates. Indeed, a composite of leather carbonized with iron oxide nanoparticles also displayed magnetic properties [84]. When used for the removal of pollutants in water remediation, magnetic properties conveniently allow for the adsorbent separation from the aqueous media [85].

## 5. Applications

**Figure 4:**
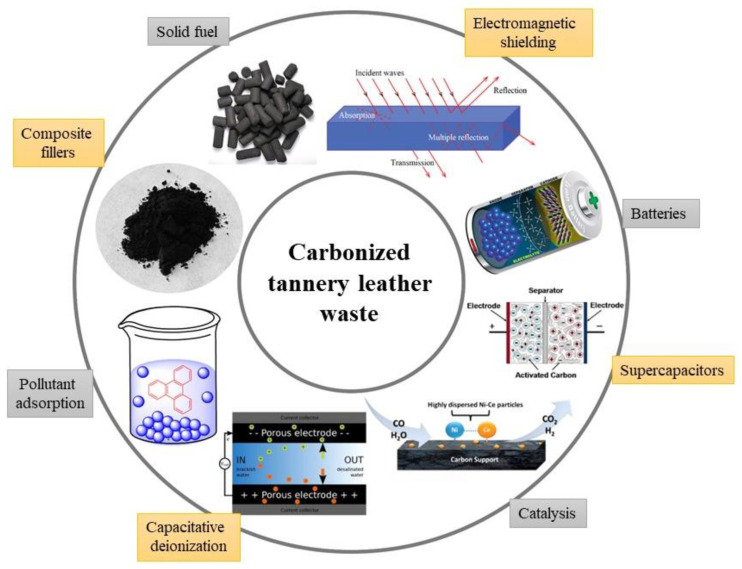
Various applications of carbonized leather waste proposed in the literature.

The application of carbonized leather waste proposed in the literature (Figure 4) is similar to the uses of other carbonized biomaterials. In contrast, however, some specific features can be exploited. These are represented by a fibrous microstructure, nitrogen content, the presence of chromium, and high electrical conductivity, as illustrated below.

### 5.1. Dye Adsorption

**Figure 5:**
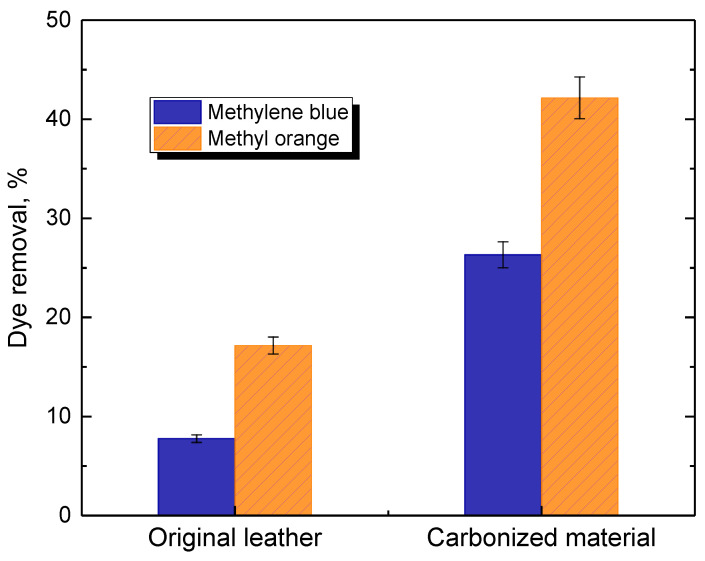
Adsorption performance of original leather compared with leather carbonized at 800 °C for the removal of methylene blue and methyl orange dyes from aqueous media at pH 5.5; adsorbent dosage 500 mg L^−1^ and dye concentration 100 mg L^−1^.

The biochar-based materials and their application in the removal of organic contaminants, viz. organic dyes, from aqueous media have been reviewed [86,87,88,89]. The ability of leather to adsorb cationic methylene blue and anionic methyl orange increases after carbonization (Figure 5). When the carbonized leather is used without subsequent treatment in aqueous media as an adsorbent, spontaneous demineralization may take place with an associated increase in the specific surface area [52] and consequent improvement in adsorption capacity. In the majority of cases, the activated carbons have been used for this purpose because the enhanced specific surface area is expected to increase the adsorption capacity.

Organic dyes have been used in adsorption experiments because their removal from the contaminated medium is easily followed by UV-vis spectroscopy [85]. Carbonized leather wastes have been applied mainly for the adsorption of a cationic dye, methylene blue (Basic Blue 9) [19,47,48,53,67,68,78], and in a single case of safranin (Basic Red 2) [90]. In the latter case, the adsorption capacity substantially depended on the type of leather precursor. The catalytic decomposition of methylene blue assisted by residual chromium compounds has also been proposed as a possible dye removal mechanism [78]. From anionic dyes, the adsorption of Acid Black 210 [66,91] and Acid Brown 414 [90] has been studied. FTIR spectroscopy has revealed dye adsorption [90]. The authors generally concluded that carbonized leathers are better adsorbents than commercially activated carbons or other carbonized substrates, e.g., sawdust [19]. Thus, carbonized leathers are potential adsorbents of organic dyes and can be applied in the pollution treatment of industrial wastewaters. In this direction, they are close to conducting polymers, which have been used for the same purpose [92,93].

### 5.2. Other Adsorptions

Carbonized leather waste has also been proposed for the removal of aromatic organic pollutants, such as acetanilide, aniline, phenol and related compounds [57,60], and tannic acid [21]. In the adsorption of gases, e.g., carbon dioxide, carbonized leather has provided better results than commercial activated carbon [54].

From the inorganic realm, the removal of chromium(VI) ions from wastewater by carbonized leathers has been reported [48,62,94]. The adsorption of heavy metal cations (lead, copper, nickel, and cobalt) has also been demonstrated [37]. The material prepared by hydrothermal carbonization has been applied in the collection of uranyl nitrate by selective adsorption [16]. Finally, the adsorption of siloxane has been investigated [61].

### 5.3. Deionization

Capacitative deionization of water is another emerging technology for water pollution treatment [69]. The method applies a potential difference over two electrodes based on the carbonized material. Electrochemical demineralization of water is used in water desalination. The electronic conductivity of electrodes is required but its level has not been quantitatively determined.

### 5.4. Supercapacitors

Materials for energy storage using biochars, viz. supercapacitor electrodes, have also been proposed [95,96]. Carbonized leather waste has displayed high faradaic pseudocapacitance in acidic medium, 268 F g^−1^ [58], or 273 F g^−1^ [73] in 0.5 M sulfuric acid. The specific capacitances 336 F g^−1^ [59], 421 F g^−1^ [63], or 550 F g^−1^ [7] in 6 M potassium hydroxide have also been reported. Finally, the specific capacitances 1800 F g^−1^ in 1 M potassium hydroxide [20] and 1960 F g^−1^ in 1 M potassium chloride [55] have been reported for activated leather-derived carbons [55]. Most of these papers mentioned good stability during charge/discharge cycling. The specific capacitances are comparable with carbon-based materials [97] and inorganic composites used in supercapacitors [98].

### 5.5. Batteries

Energy storage devices can also exploit carbonized materials. A leather waste carbonized at 1000 °C for 8 h has been used as a positive electrode in Li-ion battery with a specific capacity of 400 mAh g^−1^, which was reduced to 327 mAh g^−1^ after 50 charging cycles [22]. A charge/discharge capacity of 534 mAh g^−1^ being retained after 150 cycles is another example of this application [60].

### 5.6. Fuels

The char has also been tested as a solid fuel due to its high caloric capacity and low ash content [10] or as an energy source in metallurgy [99]. Hydrothermal carbonization, carried out in aqueous medium at 180–200 °C, led also to materials proposed as solid fuels [17], similar to a material obtained by the carbonization at 300–500 °C [18], i.e. at relatively low carbonization temperatures.

### 5.7. Electromagnetic Interference Shielding

Electromagnetic radiation shielding, especially in the GHz frequency region, is used for the protection of sensitive electronics, prevention of radiation emanation, and in stealth technologies. The conductivity of composite components is required. The lightweight carbonized aerogels of milled leather/poly(vinyl alcohol) have been offered as a shielding material for the X-band frequencies [26]. The low density of conducting carbonized leather is a benefit compared to currently used metals and inorganic oxides.

### 5.8. Catalysis

Carbonized leather waste has been tested as a metal-free electrocatalyst for oxygen oxidation reaction in an alkaline fuel cell electrode [56,72] and oxygen reduction reaction in the electrochemical generation of hydrogen peroxide [68]. Carbonized leather wastes prove to be an efficient photocatalyst in the removal of phenol from water due to the presence of chromium(III) oxide [15], despite their small specific surface area and low porosity. Ruthenium supported on carbonized leather has been used as a catalyst for the selective hydrogenation of 5-hydroxymethylfurfural [100].

### 5.9. Composite Fillers

Carbonized leather is usually handled as a fine powder. For many practical uses, it has to be combined with components that provide material properties. Carbonized leather has been used as a filler in lightweight constructional cement materials [101], an additive to mortar in construction materials [102] or bitumen binder [70,71], and as an alternative for recycled plastics [103].

Biochar has been used in agriculture as a compost additive [104]. The adsorption ability biochar derived from leather waste has been enriched with cationic micronutrients and applied as a soil conditioner [82].

## 6. Conductivity

Electrical conductivity is an important property of carbonized leather waste that has been implicitly exploited in various applications without quantitative assessment of its level. The fibrous structure of biochar is of importance in providing a sponge-like structure and the associated connective pathways that are the prerequisite for good macroscopic electrical conduction. The use of carbonized leather in supercapacitor electrodes [7,55,58,59,60,63,69] or batteries [22,60] implicitly assumes a certain level of sample conductivity. This is also true for the electrodes in electrocatalysis [56,68,72]. The conductivity of carbonized leather waste may become of importance when absorbed microwaves afford the heating during carbonization [23] or when used for electromagnetic interference shielding [26].

**Figure 6:**
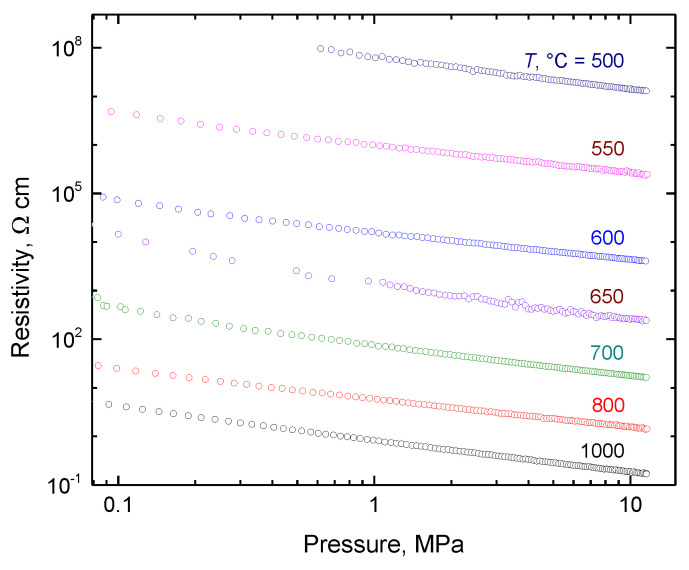
The dependence of resistivity on pressure for leather carbonized at various temperatures, *T*.

**Figure 7:**
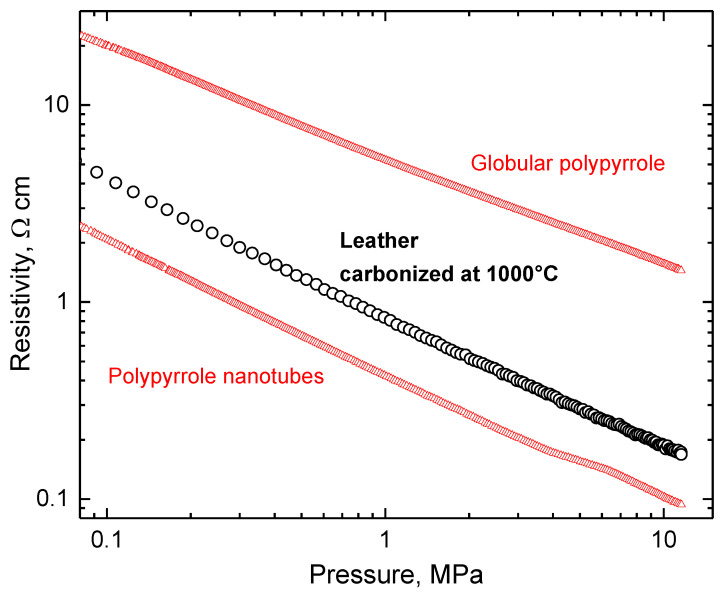
The dependence of resistivity on pressure for leather carbonized at 1000 °C (circles) compared with globular polypyrrole (PPy) and polypyrrole nanotubes (triangles).

In this section, the preliminary electrical properties of leathers carbonized in the 500–1000 °C temperature range are reported to illustrate the preparation of carbonaceous materials with conductivity suitable for various applications. The determination of conductivity, or its reciprocal value, resistivity, meets some specific requirements. Chromium-tanned beige pigskin leather is used to simulate waste and carbonized at elevated temperatures in an inert atmosphere, and the products are ball-milled. The resulting powders cannot be compressed to a solid pellet needed for the routine four-probe resistivity measurement. For this reason, the samples are placed in a glass tube and defined pressure is applied with a glass piston that carries four platinum/rhodium point electrodes on its perimeter [75]. The measured resistivity (not to be confused with resistance!) decreases with increasing carbonization temperature (Figure 6) and is reduced for the individual samples with applied pressure and consequent compression. The dependences in a double-logarithmic presentation are practically linear in the 0.1–10 MPa pressure range. Similar trends have been obtained for the macroporous melamine sponges coated with a conducting polymer, polypyrrole [105]. The resistivity behaviour of leather carbonized at the highest temperature, 1000 °C, is even closer to the conducting polypyrrole powders, globular form or nanotubes, determined under the same experimental conditions (Figure 7).

**Figure 8:**
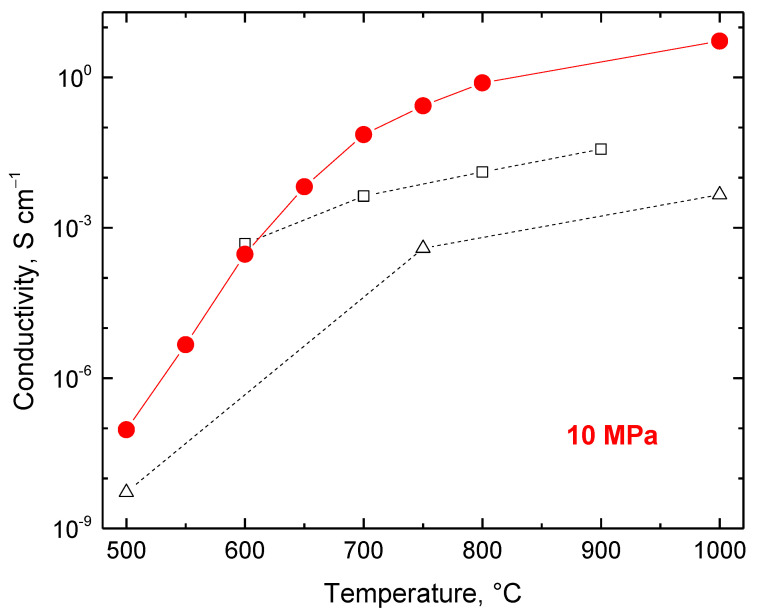
The conductivity of leather carbonized at various temperatures and determined under 10 MPa pressure (circles). The data reported in the literature for samples compressed at unspecified pressure [22] (triangles) and at 0.166 MPa [80] (squares) are included for comparison.

The conductivity (i.e. the reciprocal resistivity) of the powders measured at fixed 10 MPa pressure steadily increases from 9.4 × 10^−8^ S cm^−1^ for carbonization at 500 °C to 5.3 S cm^−1^ when treated at 1000 °C, i.e. by *ca* eight orders of magnitude (Figure 8). The conductivity of the last sample is comparable with the conductivity of the conducing polymers, e.g., powders of polypyrrole globules, 0.3 S cm^−1^, and nanotubes, 9 S cm^−1^, determined under the same pressure (the conductivity of polypyrrole compressed to a pellet at 527 MPa is still higher, 2.5 and 38 S cm^−1^, respectively [83]).

As mentioned above, the electrical properties of carbonized leather have been reported at quantitative levels only rarely. Conductivity of the waste treated at 500, 750, and 1000 °C for 8 h has been estimated as 4.7 × 10^−3^ S cm^−1^ at the highest temperature determined by the two-point method on powder compressed between two electrodes under unspecified but probably low pressure [22] (Figure 8). Such a two-point method provides a good estimate of conductivity but physicists prefer a more rigorous four-point method with separated pairs of voltage and current electrodes. The latter setup ensures a homogeneous electric field between the electrodes. In a more detailed study, the conductivity of carbonized leather was estimated again by the two-point method at 611 kPa and reached 4.8 × 10^−4^, 4.3 × 10^−3^, 1.3 × 10^−2^ and 3.7 × 10^−2^ S cm^−1^ for the samples carbonized at 600, 700, 800, and 900 °C, respectively [80] (Figure 8). The values of conductivity reported in the literature are lower than the data reported here due to the lower pressures used for the compression of powders (Figure 7); otherwise, they are in good agreement considering the various sources of leather waste.

## 7. Conclusions and Outlook

Leather waste is available in vast amounts. The carbonization of leather is an energy-consuming process and the cost of nitrogen-containing carbon production has to be balanced with the environmental profit gained by the waste conversion and the benefit of industrial application. The fact that improved conductivity of the samples obtained at increased carbonization temperature is associated with a lower yield has to be considered in production and a compromise should be sought. The presence of nitrogen atoms in the products is a value-added property compared to common activated carbons. The high content of chromium in most of the tanned leathers is an additional distinction of potential use in catalysis.

The carbonized leathers are conducting at levels comparable with conducting polymers, such as polypyrrole. This fact has not been previously mentioned in the literature despite its potential importance for applications involving the electrodes of energy-storage devices or in electromagnetic interference shielding. In addition, the adsorption and electrocatalytic decomposition of water-pollutant organic dyes can be controlled by applied electrical potential. In applications requiring just electrical conductivity, they may be considered as economic competitors of conducting polymers. Examples are conducting composites that can be applied as elements heated by passing currents or microwaves. There is a general consensus that leather waste can be converted to useful products displaying an array of value-added properties, and that some are still to be discovered.

The observation that conducting polymers and carbonized leather may have comparable conductivity opens new research directions, with the preparation of composites comprising both components. Such composites may display synergistic effects originating from the interfacial interactions of both materials or by the combination of their properties. The composites have been extensively used in supercapacitors [106], where carbonaceous materials have been modified with conducting polymers that display mixed electronic and ionic conductivity and thus facilitate electron–ion transfers. The carbons are stable with respect to charge–discharge cycles and may improve the cycling stability of conducting polymers when used in combination. A similar principle applies when the conductivity sensitivity to pH needs to be reduced.

When applied in dye removal in water-pollution treatments, the carbonaceous materials provide a high specific surface area for adsorption, while organic dyes are bound at conducting polymers by electrostatic and π–π interactions and by hydrogen bonding [85,92]. The combination of both removal mechanisms thus justifies the preparation of composite materials.

When noble-metal nanoparticles are needed to be deposited on carbon carriers in the preparation of electrocatalysts, the coating with a conducting polymer and its subsequent use for the reduction of noble-metal compounds to corresponding metals is an easy solution [107]. Carbons obtained by the pyrolysis of leather have not been so far tested as components in the composites with conducting polymers but their application potential as outlined above is obvious.

## Data Availability

Not applicable.
